# Spatial heterogeneity of parasite co-infection: Determinants and geostatistical prediction at regional scales

**DOI:** 10.1016/j.ijpara.2008.10.014

**Published:** 2009-04

**Authors:** Simon Brooker, Archie C.A. Clements

**Affiliations:** a Department of Infectious and Tropical Diseases, London School of Hygiene and Tropical Medicine, Keppel Street, London WC1E 7HT, UK; b Malaria Public Health and Epidemiology Group, KEMRI-Wellcome Trust Collaborative Programme, Nairobi, Kenya; c School of Population Health, University of Qld., Herston, Brisbane, Australia; d Australian Centre for International and Tropical Health, Queensland Institute of Medical Research, Herston, Brisbane, Queensland, Australia

**Keywords:** Parasite co-infection, Helminths, Hookworm, *Schistosoma mansoni*, Bayesian geostatistics, Control programmes

## Abstract

Multiple parasite infections are widespread in the developing world and understanding their geographical distribution is important for spatial targeting of differing intervention packages. We investigated the spatial epidemiology of mono- and co-infection with helminth parasites in East Africa and developed a geostatistical model to predict infection risk. The data used for the analysis were taken from standardised school surveys of *Schistosoma mansoni* and hookworm (*Ancylostoma duodenale*/*Necator americanus*) carried out between 1999 and 2005 in East Africa. Prevalence of mono- and co-infection was modelled using satellite-derived environmental and demographic variables as potential predictors. A Bayesian multi-nominal geostatistical model was developed for each infection category for producing maps of predicted co-infection risk. We show that heterogeneities in co-infection with *S. mansoni* and hookworm are influenced primarily by the distribution of *S. mansoni*, rather than the distribution of hookworm, and that temperature, elevation and distance to large water bodies are reliable predictors of the spatial large-scale distribution of co-infection. On the basis of these results, we developed a validated geostatistical model of the distribution of co-infection at a scale that is relevant for planning regional disease control efforts that simultaneously target multiple parasite species.

## 1 Introduction

The heterogeneities involved in the transmission dynamics of parasitic diseases are well characterised and include aggregated distributions within host populations and marked spatial heterogeneity of infection (Anderson and May, 1985; Woolhouse et al., 1997; Smith et al., 2005). Such heterogeneities are influenced by multiple factors ranging from individual (genetic and/or behavioural) via household (demographic and socio-economic) to climatic and environmental influences. Recent studies, employing a combination of geographical information systems (GIS), remote sensing, geostatistics and mathematical modelling, have proven helpful in characterising the spatial spread of infectious diseases (Russell et al., 2005; Riley, 2007) and in determining linkages between spatial patterns and climatic and environmental factors (Raso et al., 2005; Clements et al., 2006a; Danson et al., 2006; Gemperli et al., 2006; Brooker, 2007; Diggle et al., 2007). This work has yielded new insights into the epidemiology and ecology of parasitic diseases at large geographical scales that had been difficult to address with traditional approaches. The work has also allowed disease distributions to be predicted robustly and helped inform where interventions should be geographically targeted on the basis of need and potential benefit (Brooker et al., 2006a; Clements et al., 2006b; Brooker, 2007).

However, infectious agents rarely occur in isolation, with co-infection with multiple species within host populations the norm (Petney and Andrews, 1998). Co-infection refers to a situation in which individuals harbour two infections simultaneously; this differs from mono-infection in which individuals harbour only one infection (Raso et al., 2007). It is being increasingly recognised that co-infection has important ecological (Graham, 2008), epidemiological and clinical consequences (Cox, 2001; Mwangi et al., 2006; Pullan and Brooker, 2008). An ability to predict the large-scale geographical distribution of co-infections will have important implications for the design of disease control programmes. This is particularly true for integrated control programmes which simultaneously target multiple tropical diseases (Brooker and Utzinger, 2007; Hotez et al., 2007; WHO, 2007). Until recently, however, the spatial modelling of co-infection was largely ignored. Recent research has investigated the spatial distributions of co-infection with tuberculosis and HIV (Rodrigues et al., 2006) and *Schistosoma mansoni* and hookworm (Raso et al., 2006) as well as the co-endemicity of malaria and hookworm (Brooker et al., 2006b) and co-occurrence of diarrhoeal diseases and pneumonia (Fenn et al., 2005), and geostatistical models have been used to predict co-infection at local scales (Raso et al., 2006). To our knowledge, there have been no studies that have investigated the geography of co-infection at spatial scales relevant to planning large-scale control programmes, despite recent emphasis given to the distributional mapping of co-endemicity and co-infection (WHO, 2007).

In this paper we analyse empirical data from East Africa to investigate the heterogeneities and ecological correlates of mono- and co-infection with *S. mansoni* and hookworm (*Ancylostoma duodenale*/*Necator americanus*) among schoolchildren. We develop a Bayesian multi-nominal, geostatistical model of the geographical distribution of co-infection, thereby helping inform planning of integrated disease control efforts in the region.

## 2 Materials and methods

### 2.1 Data sources: outcome variable

Spatially referenced parasitological datasets were collated into a single database. Individual-level data were obtained in four separate school-based surveys conducted between 1999 and 2005 in a large, spatially contiguous area of East Africa, approximately 1260 × 590 km, incorporating all of Uganda (Brooker et al., 2004; Kabatereine et al., 2004) and parts of northwest Tanzania (Clements et al., 2006b) and southwest Kenya (Brooker et al., 2001; Koukounari et al., 2008). No previous mass treatment with anthelmintic drugs had been undertaken in any of the schools, and although seasonal dynamics in transmission stages may occur, such fluctuations may be of little significance to the overall parasite equilibrium within communities. This is because the life-span of adult worms is typically much longer (1–10 years) than the periods in the year during which the basic reproductive number (*R*_o_) is less than unity, and *R*_o_ will on average be greater than one, maintaining overall endemicity. For this reason, spatial variability in long-term synoptic environmental factors will have a greater influence on transmission success and patterns of helminth infection than seasonal variability in a location.

Detailed descriptions of survey design and study methods are provided in the original references (Brooker et al., 2001; Kabatereine et al., 2004; Clements et al., 2006b; Koukounari et al., 2008). In brief, stool samples were collected from each schoolchild and processed using standard parasitological methods. Presence of infection was based on the microscopic examination of two Kato–Katz thick smears made from a single stool specimen – a child was considered positive for *S. mansoni* or hookworm if at least one egg was detected on examination of either smear. Due to the coprological method used in the included surveys, the two species of hookworm (*N. americanus* and *A. duodenale*) were not able to be distinguished. However, the few coprological surveys in East Africa which have undertaken differential diagnosis indicate that although both species can occur, there is a predominance of *N. americanus* in the region (Sturrock, 1966a,b; Chunge et al., 1986; Ashford et al., 1992; Stoltzfus et al., 1997). The outcome variable was infection status grouped into four categories: (i) no infection; (ii) *S. mansoni* mono-infection; (iii) hookworm mono-infection; and (iv) co-infection with *S. mansoni* and hookworm.

### 2.2 Data sources: demography and environmental variables

Information on age and sex was collected by interview and confirmed against school registers. Age was categorised into approximately equally sized groups: ⩽8, 9–10, 11–13 and ⩾14 years. The geographical position of schools was determined using different global positioning systems. A range of environmental data were collated including: satellite-derived mean land surface temperature (LST) and normalised difference vegetation index (NDVI) for 1982–2000, obtained from the National Oceanographic and Atmospheric Administration’s Advanced Very High Resolution Radiometer; elevation, obtained from an interpolated digital elevation model from the Global Land Information System (GLIS) of the United States Geological Survey (Hay et al., 2006); distance to perennial inland water bodies; and degree of urbanisation (urban, peri-urban, rural and extreme rural) (Hay et al., 2005), with urban and peri-urban classes grouped into a single class due to the small size of urban areas relative to the study area. The values for each of the environmental variables at the location of each of the schools surveyed were calculated in the GIS ArcView version 9 (ESRI, Redlands, CA, USA).

### 2.3 Ethical approval

Ethical approval was obtained from the following ethics review boards: National Institute of Medical Research, Tanzania; National Health Service Local Research Ethics Committee of St Mary’ Hospital, London; Ministry of Health, Uganda; Kenyatta National Hospital, Kenya; London School of Hygiene and Tropical Medicine. In the Kenyan studies, meetings were held in participating schools prior to the surveys to explain the nature and purpose of the study to parents or legal guardians, and written informed consent was obtained. In the Tanzanian and Ugandan studies, passive consent was sought, whereby parents were informed of the study prior to the school visit through parent–teacher association meetings, when they had the opportunity to ask questions about the study, were told that participation was voluntary and that they could withdraw their child from the study at any time. In all of the studies, verbal assent to participate in the survey was also sought from the child at the time of sampling. Following the surveys, all children received treatment with albendazole (400 mg). Treatment with praziquantel (40 mg/kg) was provided only to children found to be infected with schistosomiasis or enrolled in schools where the overall prevalence was ⩾50%.

### 2.4 Statistical analysis

Heterogeneity was initially assessed on the basis of the frequency distributions of parasite prevalence. Maps of prevalence were created in GIS ArcView version 9 (ESRI, Redlands, CA, USA). Subsequently, univariate multinomial regression models were developed in Stata version 10 (Stata, College Station, TX, USA) to investigate the relationship between the outcome variable and covariates. Covariates significant at a 0.2 level were included in a Bayesian spatial multinomial logistic regression model. This model was based on the principle of model-based geostatistics (Diggle et al., 1998), where the model has two components: a deterministic component consisting of school-level climatic and individual-level fixed effects; and a stochastic component based on a stationary geostatistical model of the spatial covariance structure. Separate regression coefficients and spatial autocorrelation parameters were estimated for each of the outcome categories.

The model was developed in WinBUGS version 14 (MRC Biostatistics Unit, Cambridge, UK). The individual data were aggregated into age and gender groups and by location. Using four infection outcome groups (1 = no infection, 2 = *S. mansoni* mono-infection, 3 = hookworm mono-infection and 4 = *S. mansoni*–hookworm co-infection), we assumed that

$$\begin{array}{cc} & Y_{\mathrm{ijk}}∼\text{Multinomial}(p_{\mathrm{ijk}}\text{,}n_{\mathrm{ijk}})\text{,}\\ & p_{\mathrm{ijk}}=\frac{\phi_{\mathrm{ijk}}}{\underset{k}{\sum }n_{\mathrm{ijk}}}\text{,} \end{array}$$

where *Y_ijk_* is the observed number positive, *n_ijk_* is the number tested and *p_ijk_* is the probability of infection at location *i*, in age–gender group *j*, infection outcome group *k*, where *φ_ij_*_1_ was constrained to equal one, and for the other outcome groups

$$\log(\phi_{\mathrm{ijk}})=\alpha_{k}+\overset{T}{\underset{N=1\text{,}k}{\sum }}\beta_{\mathrm{Nk}}\times x_{\mathrm{Nijk}}+\theta_{\mathrm{ik}}\text{,}$$

where *α_k_* is the outcome group-specific intercept, $\sum_{N=1\text{,}k}^{T}\beta_{\mathrm{Nk}}\times x_{\mathrm{Nijk}}$ is a vector of *T* covariates with outcome group-specific coefficients and *θ_ik_* are outcome group-specific geostatistical random effects defined by an isotropic powered exponential spatial correlation function

$$f(d_{\mathrm{ij}}\text{;}\phi_{\mathrm{ijk}})=\exp\lfloor -(\phi_{\mathrm{ijk}}d_{\mathrm{ij}})\rfloor \text{,}$$

where *d_ij_* are the distances between pairs of points *i* and *j*, and ϕ is the rate of decline of spatial correlation per unit of distance. Non-informative priors were specified for the intercepts (uniform prior with bounds −∞ and ∞ and the coefficients (normal prior with mean = 0 and precision = 1 × 10^−4^). The prior distribution of ϕ was also uniform with upper and lower bounds set at 0.06 and 50. The *θ_ik_* were given a non-informative prior gamma distribution.

Three chains of the models were run consecutively. A burn-in of 1000 iterations was allowed, followed by 10,000 iterations where values for the intercept and coefficients were stored. Diagnostic tests for convergence of the stored variables were undertaken, including visual examination of history and density plots of the three chains. Convergence was successfully achieved after 10,000 iterations and the posterior distributions of model parameters were combined across the three chains and summarised using descriptive statistics.

Samples from the posterior distributions of the coefficients from the model were used to produce prediction maps of co-infection on a 0.2 × 0.2 decimal degree (approximately 24.4 × 24.4 km) grid covering the study area. This was done in WinBUGS using the spatial.unipred command, which implements an interpolation function (kriging, a method which minimises the variance of the predictions based on the spatial correlation function), in our case for the geostatistical random effects for each infection outcome. Predicted prevalences were calculated by adding the interpolated random effect to the sum of the products of the coefficients for the covariates and the values of the covariates at the prediction locations. For the individual-level fixed effects (age and sex), separate calculations were done, where the coefficient for the relevant age group and sex were added to the sum. The overall sum was then back-transformed from the logit scale to the prevalence scale, giving prediction surfaces for prevalence of each type of helminth infection in each age group and sex.

### 2.5 Model validation

Validation of predicted school-level prevalence of co-infection was undertaken by partitioning the data into four random sets and running the model using three of the four sets and validating the model with the remaining set. Four separate models were run using different combinations of three training sets and one validation set. The accuracy of the prediction was determined in terms of sensitivity and specificity and by the area under curve (AUC) of a receiver-operating characteristic (ROC) curve to determine the ability of the model predictions to discriminate between a true prevalence of 0% versus >0%, <10% versus ⩾10%, and <20% versus ⩾20%. As a general rule, an AUC between 0.5 and 0.7 indicates a poor discriminative capacity; 0.7–0.9 indicate a reasonable capacity; and >0.9 indicate a very good capacity. Four different estimates of AUC were derived, and an average AUC was presented.

## 3 Results

**Fig. 1:**
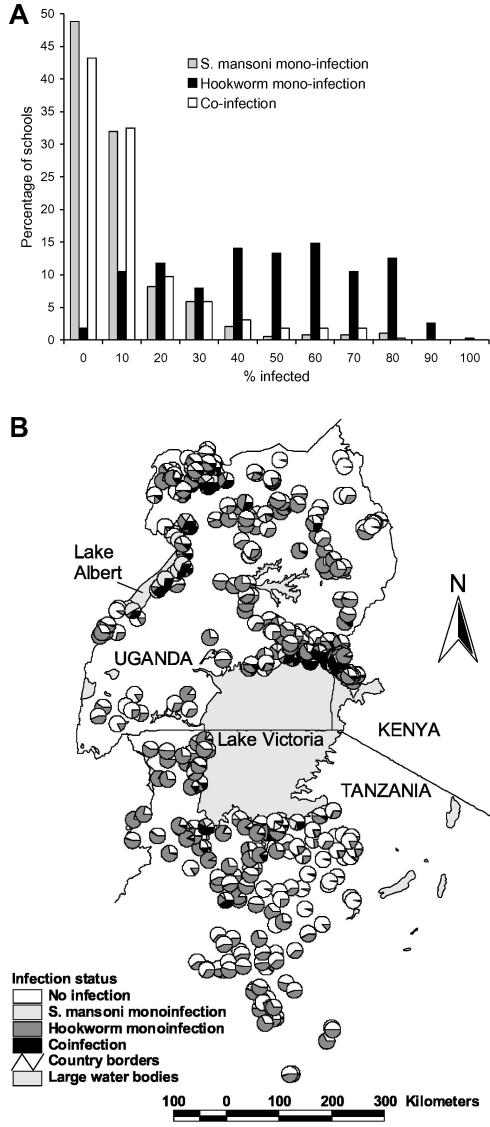
Observed heterogeneity of parasite co-infection among schoolchildren in East Africa. (A) Frequency distribution and (B) geographical distribution of mono- and co-infection with *Schistosoma mansoni* and hookworm among 27,729 schoolchildren from 395 schools in East Africa.

Data on both *S. mansoni* and hookworm were available for 27,729 schoolchildren from 395 schools. Overall, 8.1% of children were infected with only *S. mansoni* infection, 40.5% of children had only hookworm infection and 10.5% of children harboured both infections concurrently. Infection patterns were broadly similar between the sexes but males were more likely to be co-infected compared to females (11.6% versus 9.4%, Pearson *χ*^2^ = 35.7, *P* < 0.001). Patterns of co-infection differed markedly between study areas (Table 1) and between schools (Fig. 1A). The frequency distribution of *S. mansoni* mono-infection was highly skewed, with 80% of schools having a prevalence <10%. By contrast, the distribution of hookworm mono-infection was generally symmetrical, with most schools having prevalences between 40% and 69.9%. Following the pattern of *S. mansoni* mono-infection, the frequency distribution of co-infection was also highly skewed. Fig. 1B presents the geographical distribution of mono- and co-infection by school and shows that co-infection prevalence was highest along the shores of Lakes Victoria and Albert.

Table 2 presents the results of the geostatistical Bayesian multinomial logistic regression model. The rate of decline in spatial correlation *φ* and the variance of the geostatistical random effect were more similar for *S. mansoni* mono- and co-infection than for hookworm mono- and co-infection. The slightly lower (ϕ) for *S. mansoni* mono- and co-infection indicated that spatial correlation occurred over longer distances than hookworm mono-infection prevalence (i.e., spatial clusters were slightly larger). Additionally, the much higher variance of the geostatistical random effect for *S. mansoni* mono- and co-infection indicated a higher tendency towards spatial clustering than for hookworm. In other words, there was stronger evidence for spatial variability of *S. mansoni* mono- and co-infection than for hookworm mono-infection.

Individual-level and environmental variables were significantly associated with the risk of each type of infection. Risk increased with age and was lower for females. Negative associations were observed between *S. mansoni* mono-infection and elevation, distance to permanent water body, and rural areas (category 3). Risk of hookworm mono-infection was negatively associated with LST and elevation. Finally, all of the included covariates were associated with co-infection, except rural (category 3). By using a true co-infection prevalence threshold of >0% (i.e., the ability to predict the occurrence of co-infection), the average AUC of the four validation runs was 0.88. Using a true prevalence threshold of >10%, the average AUC was 0.92, and for a prevalence threshold of >20%, the AUC was 0.93.

**Fig. 2:**
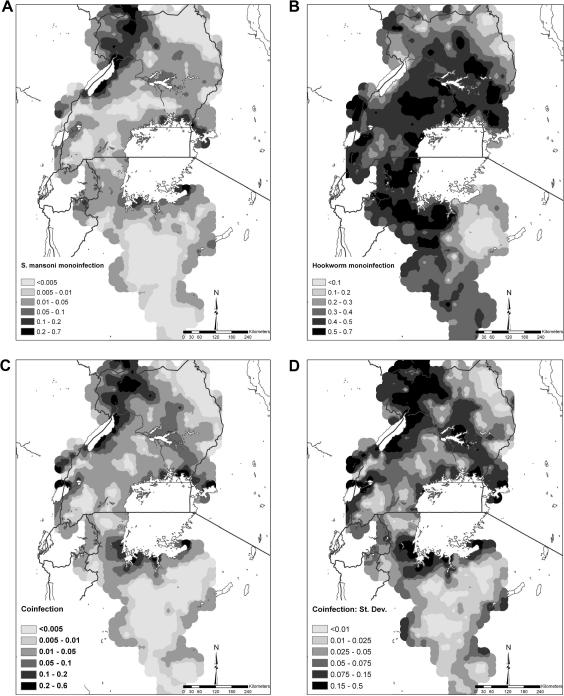
Predicted distribution of (A) *Schistosoma mansoni* mono-infection, (B) hookworm mono-infection, (C) *S. mansoni*–hookworm co-infection, and (D) SD of the predicted *S. mansoni*–hookworm co-infection among schoolchildren in East Africa. Note the different legend categories for each map for presentation purposes.

On the basis of these models and validation, it was possible to predict the distributions of mono- and co-infection (Fig. 2). The prevalence of *S. mansoni* mono-infection was greatest along the shores of Lake Victoria, Lake Albert and the Albert Nile (Fig. 2A). The predicted prevalence of hookworm mono-infection was more homogeneously distributed, with low prevalence predicted in northeast Uganda and parts of Tanzania (Fig. 2B). The predicted distribution of *S. mansoni*–hookworm co-infection was broadly similar to *S. mansoni* mono-infection in northwest and southeast Uganda (Fig. 2C). However, there were areas of high predicted co-infection prevalence in central Uganda and the southwestern shores of Lake Victoria. Fig. 2D presents the SD of the co-infection prediction and shows that the SD was greatest in areas of high prevalence of co-infection and in areas away from sampled locations.

**Fig. 3:**
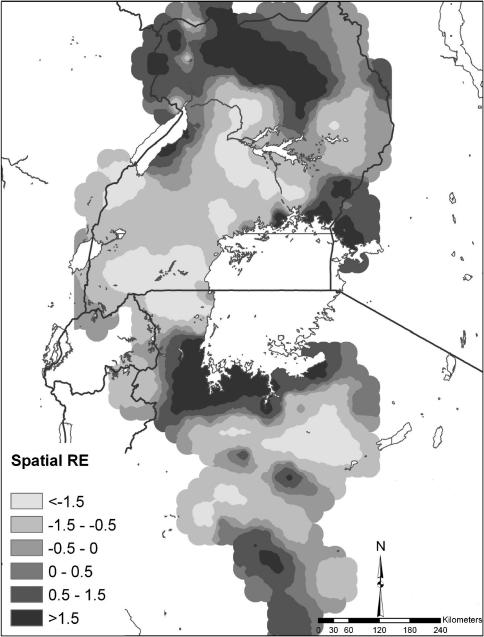
Map of the spatial random effect (RE) component of the predictions for *Schistosoma mansoni–*hookworm co-infection.

Fig. 3 shows the spatial random effect for co-infection which represents the variation in co-infection that was not explained by the model covariates yet is spatially-structured. Areas of high residual co-infection occurred in northern Uganda, western Kenya and the southwest shores of Lake Victoria.

## 4 Discussion

Biological phenomena rarely occur in isolation, and this is certainly true for parasite species within and between host populations. An ability to predict spatial distributions of co-infection will enhance our epidemiological understanding of the co-endemicity of parasite species and can provide an evidence-base for the spatial targeting of large-scale integrated control programmes (Brooker and Utzinger, 2007; WHO, 2007). Here we show it is possible to predict the regional distribution of mono- and co-infection with *S. mansoni* and with hookworm, two of the most common and geographically widespread tropical parasitic diseases, in East Africa.

Most existing studies of the spatial epidemiology of parasitic diseases focus on single species and highlight the marked spatial heterogeneity in patterns of infection (Raso et al., 2005; Brooker, 2007; Clennon et al., 2006; Sogoba et al., 2007). We demonstrate that the distribution of both *S. mansoni* mono- and co-infection is extremely focal, exhibiting a highly skewed frequency distribution and a marked spatial dependency. In contrast, the distribution of hookworm mono-infection was more symmetrical and geographically homogeneous. The generally similar patterns of *S. mansoni* mono- and co-infection suggest that the spatial distribution of co-infection is driven primarily by the distribution of *S. mansoni*, rather than the distribution of hookworm. The transmission of *S. mansoni* depends on the distribution and density of its intermediate hosts, freshwater snails. The population dynamics of snails are affected by a range of climatic and environmental factors, primarily temperature, elevation and distance to large water bodies (Sturrock, 1993; Brooker and Michael, 2000). These variables are readily incorporated within a GIS and have previously been used to predict spatial distributions of schistosomiasis (reviewed in Brooker, 2007). Our modelling shows that it is also possible to predict spatial patterns of co-infection on the basis of key climatic and environmental variables (Fig. 2C).

There were some interesting exceptions to the role of *S. mansoni* in co-infection dynamics, especially in central Uganda where the high co-infection prevalence arises from the high prevalence of hookworm. This may reflect the differential environmental determinants of each parasite species in different parts of the study area. It is also worth emphasising that the areas of high residual co-infection (Fig. 3) highlight potentially important, unmeasured covariates, which may influence transmission patterns. These might include socio-economic status, which has shown previously to be associated with *S. mansoni*–hookworm co-infection at small geographical scales (Raso et al., 2006). However, high-resolution poverty maps at regional scales are lacking since poverty data are available only at geographically aggregated levels and are often country-specific (Benson et al., 2005; Kristjanson et al., 2005; Noor et al., 2006). Another possible determinant of polyparasitism, which is also poorly mapped, is access to clean water and sanitation (Singer and Caldas de Castro, 2007); however, water and sanitation mapping is only starting to develop.

Our approach has a number of advantages and limitations. The Bayesian framework allows the explicit inclusion of spatial structure and reliable uncertainty estimation (Diggle et al., 1998; Clyde and George, 2004). The use of a single Kato-Katz smear to detect infection is a limitation since this approach is known to lack sensitivity and specificity, especially in the detection of light *S. manosni* infections (de Vlas and Gryseels, 1992), and multiple smears are recommended where possible (Booth et al., 2003). However, many of the surveys included in the present analysis were conducted in isolated communities over large geographical distances, employing mobile survey teams, thereby preventing the collection of multiple samples. Delays in processing samples after collection may also introduce bias, although this is more important for hookworm than for *S. mansoni* (Dacombe et al., 2007). Nonetheless, the fact that the same diagnostic approach was used in the included surveys at least makes the data comparable, if subject to the same biases.

The derived maps can help inform policy and decision making concerning large-scale parasite control strategies. Current efforts to control schistosomiasis and hookworm typically focus on the school-age population since much of the morbidity caused by helminth infections occur in this sub-population and school-based treatment delivery programmes offer major cost advantages (Bundy et al., 2006). These programmes deliver mass co-administration of praziquantel to treat schistosomiasis and benzimidazole anthelmintics (albendazole or mebendazole), to treat hookworm and other soil-transmitted helminth infections, in areas where both types of infections are prevalent (Bundy et al., 1991). There is a need, therefore, to identify the most appropriate mix of interventions in different areas according to patterns of mono- and co-infection (Bundy et al., 1991; Raso et al., 2007); blanket treatment of praziquantel and benzimidazoles may lead to treating large numbers of individuals unnecessarily. Our models were aimed at national policy and decision making, and argue for a spatially targeted approach to control of schistosomiasis and co-infection, but widespread, mass control of hookworm within the broad limits of its geographical range.

This study provides a reasonable prediction of mono- and co-infection with two of the widespread and important helminth infections that infect humans in the developing world. Our approach may well apply to other co-infections where a geographically targeted approach to control is required. For example, there has been increased advocacy for the logistic and economic benefits of integrating national control programmes targeting a range of so-called neglected tropical diseases, including schistosomiasis, soil-transmitted helminths, lymphatic filariasis and onchocerciasis (Richards et al., 2006; Hotez et al., 2007). There are also calls to investigate the potential of a combined approach to helminth-malaria control (Brooker et al., 2007). It will therefore become increasingly important to understand the main drivers of co-infection with different species. For example, although malaria and hookworm occur coincidentally over much of sub-Saharan Africa (Brooker et al., 2006b), it remains unclear whether the climatic and environmental drivers of each disease are the same, similar or spatially co-incident. In turn, there is a requirement for better empirical data on patterns of co-infection and to develop more models of co-infection for a range of tropical diseases in varying transmission settings. Finally, there is a need for spatially explicit economic models to be developed, which include estimates of programme cost and cost-effectiveness in order to support decision making.

## Figures and Tables

**Table 1:** Prevalence (% infected) of infection and mono- and co-infection with *Schistosoma mansoni* and hookworm among 27,729 schoolchildren from 395 schools in East Africa.

	Study region				Total
	Bondo, Kenya 2005	Busia, Kenya 2000	NW Tanzania 2005	Uganda 1999–2002	(range by school)
No. of children	1092	1677	8617	16,343	27,729
*S. mansoni*	14.3	22.3	4.4	26.1	18.7 (0–97.3)
Hookworm	47.2	77.1	48.0	50.2	51.0 (0–95.7)
No infection	44.9	16.5	50.3	38.0	40.9 (0–100)
*S. mansoni* mono-infection	7.9	6.4	1.7	11.8	8.1 (0–80.0)
Hookworm mono-infection	40.8	61.2	45.2	35.9	40.5 (0–91.6)
Co-infection	6.4	15.9	2.8	14.3	10.5 (0–71.2)

**Table 2:** Bayesian multinomial logistic regression model for mono- and co-infection with *Schistosoma mansoni* and hookworm with geostatistical random effects, based on parasitological data among 27,729 schoolchildren from 395 schools in East Africa.

Variable	*β* posterior mean (95% posterior CI)	OR posterior mean (95% posterior CI)
*S. mansoni mono-infection*		
Intercept	−3.838 (−4.696 to −2.888)	
Elevation	−1.050 (−1.507 to −0.550)	0.350 (0.222–0.577)
Distance to permanent water body	−1.475 (−2.353 to −0.793)	0.229 (0.095–0.452)
Urban–rural 3	−0.843 (−1.570 to −0.234)	0.430 (0.208–0.792)
Urban–rural 4	−0.477 (−1.492 to 0.367)	0.621 (0.225–1.444)
LST	−0.129 (−0.475 to 0.221)	0.879 (0.622–1.247)
Sex (female)	−0.154 (−0.269 to −0.038)	0.857 (0.764–0.963)
Age (9–10 years)	0.515 (0.315–0.721)	1.674 (1.370–2.057)
Age (11–13 years)	0.893 (0.725–1.060)	2.443 (2.064–2.886)
Age (⩾14 years)	1.055 (0.785–1.312)	2.872 (2.192–3.714)
Phi (rate of decay)	3.517 (1.727–7.214)	
Variance of spatial random effect	6.388 (3.523–11.780)	

*Hookworm mono-infection*		
Intercept	−0.636 (−1.135 to −0.323)	
Elevation	−0.260 (−0.429 to −0.113)	0.771 (0.651–0.893)
Distance to permanent water body	−0.063 (−0.277 to 0.142)	0.939 (0.758–1.153)
Urban–rural 3	−0.016 (−0.386 to 0.317)	0.984 (0.680–1.373)
Urban–rural 4	0.150 (−0.194–0.593)	1.162 (0.824–1.810)
LST	−0.513 (-0.686 to −0.330)	0.599 (0.504–0.719)
Sex (female)	−0.094 (−0.151 to −0.034)	0.910 (0.860–0.966)
Age (9–10 years)	0.153 (0.043–0.261)	1.165 (1.044–1.298)
Age (11–13 years)	0.435 (0.329–0.539)	1.545 (1.390–1.714)
Age (⩾14 years)	0.631 (0.489–0.762)	1.880 (1.630–2.143)
Phi (rate of decay)	4.980 (3.383–7.332)	
Variance of spatial random effect	1.311 (0.984–1.760)	

*S. mansoni and hookworm co-infection*		
Intercept	−4.352 (−5.024 to −3.744)	
Elevation	−1.206 (−1.626 to −0.754)	0.299 (0.197–0.471)
Distance to permanent water body	−1.203 (−1.736 to −0.546)	0.300 (0.176–0.579)
Urban–rural 3	−0.499 (−1.037 to 0.024)	0.607 (0.355–1.024)
Urban–rural 4	−0.290 (−1.165 to 0.481)	0.748 (0.312–1.618)
LST	−0.563 (−1.170 to −0.139)	0.570 (0.310–0.870)
Sex (female)	−0.364 (−0.465 to −0.259)	0.695 (0.628–0.772)
Age (9–10 years)	0.597 (0.416–0.791)	1.816 (1.516–2.206)
Age (11–13 years)	1.096 (0.937–1.259)	2.992 (2.553–3.522)
Age (⩾14 years)	1.344 (1.103–1.581)	3.834 (3.013–4.860)
Phi (rate of decay)	3.758 (2.101–7.363)	
Variance of spatial random effect	6.339 (3.977–9.954)	

CI, credible interval; LST, land surface temperature.
